# Chattanooga Medical Society

**Published:** 1894-09

**Authors:** W. C. Townes

**Affiliations:** President in the Chair


### Society Reports.

### CHATTANOOGA MEDICAL SOCIETY.

### REGULAR MEETING SEPTEMBER 7, 1894.

*President W. C. Townes in the Chair.*

Essay of the evening: "Some General Considerations in Reference to Diseases of the Eyes," by Frank Trester Smith, A.M., M.D.

Patients who present themselves with eye troubles generally complain of defective vision, of pain referred to the eyes, or of some inflammatory condition. Concerning the first, there are some facts which should be borne in mind. If there is no perception of light, nothing can be done to improve the vision, and also in those cases where the cornea is entirely opaque; but wherever there is perception of light and any clear cornea, the unfortunate should have the benefit of an expert examination. In cases of defective vision, where the eye is of normal appearance, the probabilities are that spectacles will be indicated.

Pain in the eye, as a rule, is due to neuralgia, inflammation, or glaucoma. The diagnosis between glaucoma and neuralgia is difficult sometimes, because the characteristic sign of the former, increased tension, is frequently wanting at the time of the examination, but there are generally other indications which should enable a general practitioner, without the aid of the ophthalmoscope, to make the diagnosis. The dilated pupil, the anesthetic cornea, the history of halos around a light, the fact that the patient has been compelled to change glasses frequently, all point to the nature of the affection.

It has seemed to me that it would be convenient to divide the inflammations into two classes, viz., diseases of the conjunctiva and diseases of the other structures. This classification is useful because the prognosis and treatment differ according to whether the inflammation is in the conjunctiva, or in some other part of the eye. Diseases of the conjunctiva are not dangerous so long as the cornea is not implicated. A chronic inflammation may sometimes cause much inconvenience and discomfort by the cicatricial contraction and the atrophy of the secretory glands, but so long as the cornea is unchanged, the integrity of the eye itself is unimpaired. Generally speaking, the prognosis in diseases of the other structures is more serious than in diseases of the conjunctiva. The vast majority of eye affections are those of the conjuctiva, and they will recover if not interfered with. The only danger is that in the course of the disease the other structures will be involved.

As to the diagnosis, it is not always easy. In inflammations of the cornea, iris and ciliary body, there is a secondary conjunctivitis which is too often treated as the primary diaease. However, there is one safe course, and that is, when in doubt treat the case as though it were one of iritis by the use of atropine. If the latter drug is not specially indicated, no harm will be done if you are careful to feel the eyeball for any increased tension in subjects over forty years of age.

In the treatment of eye affections, the classification I have given is especially useful, for generally speaking, in the conjunctivitides cold and astringents are indicated, while in the other inflammations, heat and atropine should be used. There are some few exceptions to this rule, for occasionally heat seems to have a better effect than cold in conjunctivitis, and in diphtheritic conjunctivitis cold would be contraindicated.

The line of thought I have developed briefly in this paper has been suggested by my work in teaching. I have endeavored to simplify the subject as much as possible, and to impress thoroughly, in the first part of the course, these general principles, and then to work in the details and exceptions later.

DR. N. C. STEEL said that when a patient has a mucous or muco-purulent discharge, the case is generally one of conjunctivitis; if a watery discharge, it is, as rule, one of keratitis or iritis. The use of atropine in the aged is dangerous, as it may induce an attack of glaucoma. Did not like to call any case neuralgia. To do so was a confession of inability to ascertain the cause of the pain.

Did not remember that he had ever had a case which he called neuralgia. Of course there are cases of pain in or about the eye, the cause of which we may not know. This may be true of any other organ or part of the body, and it is a convenient custom of doctors to call such pain neuralgia. Practically it is sometimes very difficult to differentiate between iritis and glaucoma in their early stages.

Concerning spectacles, general practitioners sometimes laugh at oculists for fitting them, considering it undignified. Some had become almost offended at him because he had charged ten dollars for fitting glasses which required ten times the scientific skill necessary to cut off a thigh. It would take him one or two hours to fit a difficult case, while the amputation could be done in a few minutes. This view of the matter, held by a few general practitioners, is a result of a misunderstanding of the nature of complicated errors of refraction and of muscle anomalies. He felt like making a speech on the importance and dignity of scientific spectacle-fitting.

DR. E. L. JONES said that disease of the frontal sinuses may cause the pain that is diagnosed as neuralgia. He would like to ask where the line of distinction was drawn between cases of follicular conjunctivitis and trachoma by those who make a distinction. To discuss a subject of such a general nature as Dr. Smith's paper, is like what Sam Jones calls trying to bite off of a pumpkin.

DR. B. S. WERT asked about the use of heat and cold. Dr. Steel, in replying, said that there were exceptions to the rules, as in a case of corneal trouble where heat would be indicated, and yet the patient could not bear heat but was relieved by cold. The use of poultices on the eyes, condemned in the books, was again coming into vogue, but must be used with discrimination. Should never be used in conjunctivitis.

Trachoma produces marked subjective symptoms, and those cases where there are elevations without such symptoms were not trachoma. Can cure nearly all cases of trachoma in one to three months with granulation forceps and Hodge's mixture as the chief remedies. Does not use copper at all. It is very painful, and trachoma can be cured without it.

DR. SMITH said that the difficulty about the use of copper was that it was improperly used, the idea being that its results were due to friction. This is a mistake. The bluestone should lightly touch the elevated places. It had seemed to him that all of those cases where the elevations resembled trachoma granules were trachoma. In the Orphans' Home here, a few years ago, there were a number of cases of this kind among the younger children—too many for it to be accidental. Among the older children there were many bad cases of trachoma. He believed that if these cases were watched and not treated, it would be found that some would recover, while others would develop a severe form of trachoma. In New York such cases were not admitted to public institutions without first having their eyes treated.

He wanted to call especial attention to the use of atropine in cases of doubtful diagnosis. He had never seen an account of a death from its use. There are occasionally cases which have an idiosyncrasy against the drug, and acute mania may be produced by small doses; such symptoms as headache, flushing of the face, and dryness of the throat are more frequent and cause no alarm, as they disappear without treatment.

DR. BERLIN related a case where a patient had taken one or one and a fourth grains. She had all the classical symptoms of atropine poisoning. He told the friend to leave her alone and she would be all right in three days. In experiments on dogs and other animals, he had never been able to kill them by atropine. Where cases of atropine poisoning have died, it has been due to the remedies used and not to the effects of the drug.

---

OCULAR MALARIA.—It is now a well recognized fact that the structures of the eye, especially the cornea and conjunctiva, are subject to malarial affections, periodical in character, differing from the usual affections of these parts, but involving actual tissue change, and amenable to quinine or other anti-malarial treatment. Some ulceration or abrasion of the corneal epithelium may occur, or intra-ocular hemorrhage during the cold stage of a paroxysm.—*Ex.*

